# Temporal Dynamics of Bacterial Communities in Soil and Leachate Water After Swine Manure Application

**DOI:** 10.3389/fmicb.2018.03197

**Published:** 2018-12-21

**Authors:** Elizabeth L. Rieke, Michelle L. Soupir, Thomas B. Moorman, Fan Yang, Adina C. Howe

**Affiliations:** ^1^Agricultural and Biosystems Engineering, Iowa State University, Ames, IA, United States; ^2^National Laboratory for Agriculture and the Environment, United States Department of Agriculture–Agricultural Research Service, Ames, IA, United States

**Keywords:** swine manure, soil, drainage water, bacterial communities, 16S rRNA

## Abstract

Application of swine manure to agricultural land allows recycling of plant nutrients, but excess nitrate, phosphorus and fecal bacteria impact surface and drainage water quality. While agronomic and water quality impacts are well studied, little is known about the impact of swine manure slurry on soil microbial communities. We applied swine manure to intact soil columns collected from plots maintained under chisel plow or no-till with corn and soybean rotation. Targeted 16S-rRNA gene sequencing was used to characterize and to identify shifts in bacterial communities in soil over 108 days after swine manure application. In addition, six simulated rainfalls were applied during this time. Drainage water from the columns and surface soil were sampled, and DNA was extracted and sequenced. Unique DNA sequences (OTU) associated with 12 orders of bacteria were responsible for the majority of OTUs stimulated by manure application. *Proteobacteria* were most prevalent, followed by *Bacteroidetes*, *Firmicutes*, *Actinobacteria*, and *Spirochaetes*. While the majority of the 12 orders decreased after day 59, relative abundances of genes associated with *Rhizobiales* and *Actinomycetales* in soil increased. Bacterial orders which were stimulated by manure application in soil had varied responses in drainage waters over the course of the experiment. We also identified a “manure-specific core” of five genera who comprised 13% of the manure community and were not significantly abundant in non-manured control soils. Of these five genera, *Clostridium sensu stricto* was the only genus which did not return to pre-manure relative abundance in soil by day 108. Our results show that enrichment responses after manure amendment could result from displacement of native soil bacteria by manure-borne bacteria during the application process or growth of native bacteria using manure-derived available nutrients.

## Introduction

Rising demands for pork products in developing countries has led to increased production in the United States (Narrod et al., 2011). In 2018, pork production in the United States is estimated to reach 27 billion pounds, increasing 2017 totals by 5% (Economic Research Service [ERS], and United States Department of Agriculture [USDA] et al., 2018). Increasing demands, coupled with production shifting toward confined animal feeding operations (CAFOs), has resulted in increased availability of manure as well as needs for its disposal (Choudhary et al., 1996). Because swine manure contains valuable nutrients, it is often applied to agricultural land as an alternative to inorganic fertilizer. Previous work has documented both benefits and risks of manure application for soil health. Among benefits, swine manure application has been observed to increase aggregate stability, bacterial diversity and soil microbial biomass and activity following application (Larkin et al., 2006; Hammesfahr et al., 2008, 2011; Zhang et al., 2012; Adesanya et al., 2016). The addition of organic nutrients through manure amendments also introduces foreign microbial communities, which could be of concern to humans, e.g., fecal-borne pathogens, to surrounding soils (Ziemer et al., 2010; Gordoncillo et al., 2013; Haack et al., 2015; Givens et al., 2016). With rainfall, manure-associated nutrients and microbial communities also are transported to downstream waters (Sharpley and Withers, 1994; Hoang et al., 2013; Luby et al., 2016).

Currently, little is understood about manure microbial communities and their response in soils after manure application. Past studies have identified significant differences between the microbial community structures of agricultural soils and manure microbiomes. Depending on location and management practices, major phyla in agricultural soils largely consist of varying combinations of *Proteobacteria, Verrucomicrobia, Acidobacteria, Chloroflexi*, and *Actinobacteria* (Trivedi et al., 2016). Conversely, *Firmicutes* and *Bacteroidetes* are the primary constituents in swine manure microbiomes (Looft et al., 2012; Lu et al., 2014; Kumari et al., 2015; Pajarillo et al., 2015). Because of the observed contrast between manure-associated and native soil microbial communities, there is a need to identify and track manure-amended microbial impacts in agricultural environments. Short-term increases in manure-associated species or operational taxonomic units (OTUs) closely related to *Proteobacteria*, *Bacteroidetes*, and *Chloroflexi* have been observed following manure amendments to soils (Ding et al., 2014; Riber et al., 2014; Leclercq et al., 2016; Liu et al., 2017). These previous inquiries provide information regarding manure’s impact on existing soil microbial communities, but lack the ability to track changes in drainage waters associated with agricultural soils.

The impacts of manure amendment to surrounding environments in United States’ Upper Midwestern agriculture, (Illinois, Iowa, Minnesota, and Wisconsin), is further amplified by the presence of artificial subsurface drainage, which can accelerate the movement of nutrients and microorganisms. Approximately one third of Iowa cropland contains subsurface drainage to artificially lower the water table, allowing access to fields and aerated soils for crop growth (Zucker and Brown, 1998). Previous studies have shown that tile drainage provides a pathway that can expedite the movement of nutrients, herbicides, pesticides, and bacteria into water (Buhler et al., 1993; Flury, 1996; Jaynes et al., 2001; Rieke et al., 2018a). However, less in known regarding the impact of manure’s microbial community on existing agricultural soil microbial communities and communities in associated subsurface drainage.

To specifically track manure-associated impacts on soils and drainage waters, we constructed soil columns to simulate the field environment. Precisely, soil columns were amended with manure and treated with simulated rainfall to assess the temporal variation in manure-associated and endemic soil microbial communities in the soil and drainage water. Using phylogenetic marker sequencing, we identified manure-derived operational taxonomic units (MDOs) and manure-stimulated operational taxonomic units (MSOs). We hypothesized manure addition (i.e., MDOs) to the soil may introduce and/or sustain manure associated microbial communities or promote growth of native soil microbes in soil and drainage waters (i.e., MSOs). The objectives of this study were to identify specific MDOs that are not abundant in soils and track the fate of MDOs and MSOs in soils and drainage waters.

## Materials and Methods

### Soil History

Soil columns under four different management practices were collected on November 6, 2015 from plots at Iowa State’s Northeast Research and Demonstration Farm, near Nashua, IA, United States (43.0° N, 92.5° W). All plots where columns were sampled were maintained in corn-soybean rotations, with nitrogen application prior to the corn growing season. Nitrogen was applied either in the form of swine manure or urea ammonium nitrate (UAN) and plots were maintained under chisel plow or no-till managements, creating four different overall management practices: manured no-till, non-manured no-till, manured chisel plow, and non-manured chisel plow. Soils at the farm are Mollisols consisting of: moderately well to poorly drained Kenyon silty–clay loam (fine-loamy, mixed, super active, mesic Typic Hapludolls), Floyd loam (fine-loamy, mixed, super active, mesic aquic Pachic Hapludolls), and Readlyn loam (fine-loamy, mixed, superactive, mesic Aquic Hapludolls) over glacial till, with 1–3% slopes (Farthelrahman et al., 2011). Manure was last injected as 10–15 cm deep bands on November 6, 2014. Manure had not been applied to control (non-manured) plots since 1978, while application rates to plots receiving manure have varied since 1993. Chisel plow tillage and no-till management were chosen due to their prevalence in Iowa agriculture. Agricultural fields under no-till management form preferential flow paths, or macropores, which may enhance leaching of bacteria (Jamieson et al., 2002). Chisel plow tillage disrupts macropores and aerates soil, creating different transport pathways for constituents through soil profiles.

### Column Construction

Thirty-six PVC pipes (15.24 cm inner diameter, 60.96 cm long) were gently pushed into the soil and pulled out using a Giddings soil probe to obtain minimally disturbed soil columns from the plots described previously (Supplementary Figure 1). Soil depths within the column ranged from 46 to 51 cm. Foam blocks were taped to the bottom of columns for transport back to Iowa State University. Column bottoms were fitted with 15.24 cm diameter PVC pipe end caps. Prior to attachment, a single hole was drilled in the middle of the end cap to insert tubing for drainage collection (Supplementary Figure 2). Caps were then filled with ASTM 20-30 Test Sand (Humboldt Mfg Co, Elgin, IL, United States) in order to create a sand–soil interface when attached. Fiberglass mesh, with 1.4 mm square sizing, was attached to the inside of caps over the drilled hole to prevent sand loss. Prior to construction (161 days following extraction) soil columns were periodically treated with 200 mL of deionized water to maintain soil moisture as described below.

### Manure Collection

The manure for this study was obtained from a commercial finishing swine facility, near the Iowa State Northeast Research Farm. The facility maintains 2.5 animal rotations per year, with manure pumped annually from a storage pit located below the animal housing. Manure samples were collected directly from the injector on November 3, 2015. Samples were stored on ice while being transported back to Iowa State University. Samples were stored at 4°C in 19 L buckets until applying on soil columns. The manure contained a moisture content of 92% and a pH of 7.5. Dry matter within the manure was comprised of 8% total nitrogen, 38% carbon, and 6% phosphorus.

### Manure Application

We simulated manure injection zones in manure-treated columns by removing a 10.2 cm diameter, 15.2 cm deep section of soil from the center of each column on day 0 of the experiment (Supplementary Figure 2). The displaced soil was homogenized by hand and subsampled in 10–20 g aliquots. The tops of non-manured control columns were also sampled at the same time by scraping off 10–20 g off the top of the column, rather than removing a 15.2 cm deep section and frozen at -20°C. Manured columns were treated with 750 mL of liquid swine manure to approximate a 7.8 cm wide, 15.24 cm deep manure injection band based on volume. After manure application, excavated soil was replaced in manured columns to obtain pre-application soil depths. Integration of excavated soil was implemented to mimic the soil–manure interface observed in field, following knifing of soil and manure injection. Manure subsamples were frozen at this at time at -20°C for DNA extractions. Soil columns were maintained in a vertical position in a constructed rack at room temperature.

### Simulated Rainfall, Effluent Capture, and Soil Sampling

Simulated rainfall events were conducted at 10, 24, 35, 59, 80, and 108 days after manure application (DAM). Single column rainfall simulators were constructed by drilling six holes in 15.24 cm diameter PVC pipe end caps to hold 20 G × 1½ in. hypodermic needles (BD Franklin Lakes, NJ, United States). One hole was drilled in the center of the cap, with the remaining five holes drilled in a circular pattern with a 5.1 cm radius from the central hole. Simulators were placed on top of each column and filled with 1 L of deionized (DI) water. Leachate was collected in 1 L sterile Nalgene bottles located on racks below the columns and processed 48 h after the beginning of each event. In order to maintain soil moisture contents representative of field conditions columns received additional wettings in between rainfall events using the individual rainfall applicators. A 1-L pan of DI water, with a 15.2 cm diameter, was placed on the top of the column rack during the experiment. At 48 h prior to rainfall events the amount of water evaporated from the pan was recorded. DI water was added at a rate of amount evaporated plus an additional 100 mL to each column in order to conservatively approximate field capacity at the time of each rainfall event. After measurement of DI volume evaporated from the pan, the pan was emptied and filled with a fresh 1 L of DI water. Additionally, this process was repeated once a week throughout the experiment during weeks without simulated rain events. Three chisel plow and three no-till columns were sacrificed for sampling 48 h after rainfall events conducted 24, 59, and 108 DAM. The top 15 cm of each column were extracted separately and mixed by hand due to moist soil conditions and high concentrations of organic matter in soils amended with manure. The homogenized soils were subsampled into 10–20 g aliquots and frozen at -20°C. Composite no-till and chisel plow soil samples from plots with a history of manure application collected on days 0, 24, 108 were analyzed for total nitrogen, total carbon, pH, and texture.

### DNA Extraction and 16S rRNA Sequencing

Column effluent was filtered through 0.22 μm sterile filters and frozen at -20°C until DNA extractions were performed using Mo Bio Power Water DNA kits. Three 50 mL aliquots were filtered from manure treated effluent from columns which were associated with the following column sacrifice. In total, 18 50 mL aliquots were collected from each rainfall event, creating an overall sum of 108 effluent samples. Soil DNA was extracted using PowerSoil 96 Well Soil DNA Isolation kits (Qiagen). Soil aliquots for each well were weighed and recorded. The target weight for each soil aliquot was 0.25 g. Two soil aliquots were used for DNA extraction from each of the 36 columns on day 0 of the experiment, prior to manure application. Additionally, two soil subsamples were collected for DNA extractions from manured treated soil columns which were destructed. In total, soil DNA extraction was performed on 180 soil samples. DNA samples above 10 μg mL^-1^ were diluted to 10 μg mL^-1^ to help negate potential effects of inhibitors and non-specific binding of primers. The V4 region of bacterial 16S rRNA gene using F515/R806 primers was amplified using methods previously described (Kozich et al., 2013). Sequencing of bacterial amplicons was performed on Illumina Miseq with Miseq Reagent Kit v2. Sequences were deposited in the NCBI SRA with the following accession number: PRJNA506065 (Supplementary Table 4).

### DNA Sequence Processing

Ribosomal Database Project (RDP) Paired-end Reads Assembler (Cole et al., 2014) was applied to assemble 150 base paired-end bacterial 16S rRNA gene sequences. Minimal and maximum assembled lengths were 250 bases (l–250) and 280 bases (-L 280). Assembled sequences with expected maximum error adjusted Q scores less than 25 over the whole sequence were removed. Chimeras *de novo* were removed with Usearch (8.1 64bit) (Edgar et al., 2011). RDP’s 16S rRNA gene training set sequences (No15) was used to eliminate chimeras of known reference genes. Resulting sequences were clustered at 97% sequence similarity by CD-HIT (4.6.1) (Fu et al., 2012), and abundances in each sample were estimated. CD-HIT was chosen due to its speed and demonstrated production of clusters which were highly similar to true numbers of OTUs derived from simulated complex data (Bonder et al., 2012; Chen et al., 2013). OTU sequences were assigned taxonomic information using RDP classifier (Wang et al., 2007) with a confidence cutoff at 50% (-c 0.5). OTUs witnessed less than five times were removed from the dataset. Additionally, manure, soil and drainage samples containing less than 6,000 sequences were removed, which resulted in 162 samples used in the experiment. Remaining samples were rarified to 6,076 sequences using the function rarify_even_depth in the R package phyloseq (McMurdie and Holmes, 2013).

### Statistical Analysis

Permutational multivariate analysis of variance using distance matrices was performed with the *adonis* function found in the R package vegan (Dixon, 2009). Differential expression analysis based on the negative binomial distribution (Gamma-Poisson) was completed using the function *Deseq* from the R package DESeq2 (Love et al., 2014) to identify OTUs whose abundances were significantly different based on selected treatments. Non-metric multidimensional scaling (NMDS) was performed using the ordinate command in *Phyloseq* (McMurdie and Holmes, 2013) with Bray–Curtis distances. Alpha diversity was calculated using the Shannon diversity index resulting from the *estimate_diversity* command in *Phyloseq.* Code for analysis is located at https://github.com/lizrieke/Nashua-Manure-Column-Study.

## Results

Our experiment resulted in a total of 3 manure, 117 soil, and 69 water samples from a total of 36 columns over 108 days sampling period. Analysis was performed to characterize physical and chemical changes following manure application (Table 1). Both total nitrogen and to carbon increased following manure application. Total carbon and nitrogen percentages remained elevated for the remaining portion of the environment. Conversely, soil pH increased following manure application, but returned to pre-manure application values by the end of the experiment. Soil texture remained largely unchanged over the course of the study.

**Table 1:** Manured soil total nitrogen, total carbon, pH, and texture of no-till and chisel plow 15 cm composite samples.

Sample day	% N	% C	pH	% Sand	% Silt	% Clay
Day 0^a^	0.22	2.42	6.1	30	40	30
Day 24	0.37	3.79	8.1	32	38	30
Day 108	0.35	3.82	5.8	30	40	30


*^*a*^Samples were taken prior to manure application.*

**FIGURE 1:**
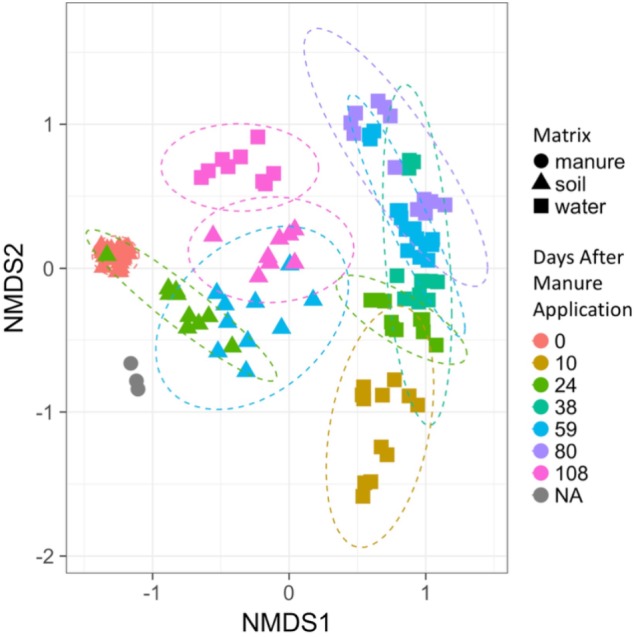
Non-metric multidimensional scaling (NMDS) of the 16S-rRNA gene sequences from manure, manure applied to soil, and water draining from manure-treated soil. *K* = 2, stress value = 0.1180937 using Bray–Curtis distances. Day 0 soil samples were collected prior to manure application. Dashed ellipses represent 95% confidence intervals around soil and water group centroids by sampling day. Manure samples are colored gray.

For each sample, the microbial community structure was determined from amplicon sequencing of the 16S rRNA gene. We observed significant differences (*p* < 0.05) in both soil and water microbial communities from control (no-manure amendments) and manure amended columns using the Adonis function in vegan (v. 2.5-3) (Oksanen et al., 2013). Overall, variation in soil and water community structures were driven by two main factors, sample matrix and the number of DAM. Permutational multivariate analysis results identified 40% of variation in effluent community structure in manured columns was derived from DAM, while DAM attributed to 56% of variation in manured soil community structures. This analysis was run separately by matrix due to the experiment’s unbalanced sampling design (four soil sampling time points and six water sampling time points). Permutational multivariate analysis was run on a subset of water and soil samples (24, 59, and 108 DAM) to ensure a balanced sampling design. Results indicated 22% of variation in soil and effluent microbial community structure was attributed to sample matrix. NMDS identified similar groupings of microbial communities based on matrix (manure, soil or drainage water) and DAM (Figure 1). Although tillage history was associated with significant differences in community structure, only 2% of variation was explained by this factor and therefore no-till and chisel plow treatment were combined for the remaining analysis.

**FIGURE 2:**
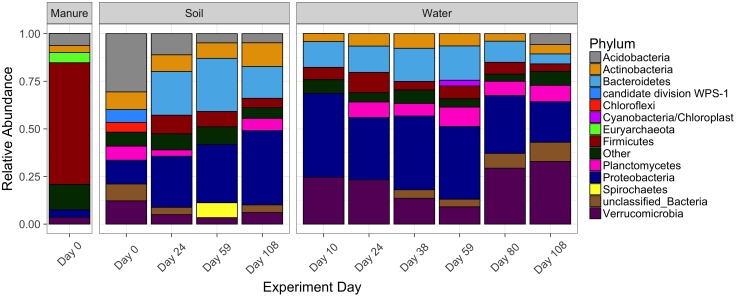
Relative abundance of sequences matching major phyla in manure, soil treated with manure and leachate water. Day 0 soil samples were collected prior to manure application from plots with a history of manure application. The other phyla grouping represent OTUs whose sequences matched known phyla, while the unclassified bacteria grouping represents relative abundances of OTUs which did not match known phyla.

The major phyla comprising manure and soil microbial communities prior to manure amendment were characterized. Overall, manure was comprised primarily of *Firmicutes*, contributing to 64% of total relative abundances of the total microbial community (Figure 2). The remaining 36% of OTUs were associated with *Acidobacteria, Euryarchaeota, Proteobacteria, Actinobacteria, Verrucomicrobia*, and *Bacteroidetes*. Unamended soil microbial communities (DAM 0) relative to manure communities were more diverse, comprised mainly of *Acidobacteria, Actinobacteria*, *Proteobacteria*, and *Verrucomicrobia*. Unamended soil microbial communities contained an average a Shannon diversity index of 6.1 ± 0.21, compared to an average of 4.9 ± 0.141 in manure. After manure amendment, we observed various shifts in the relative abundances of multiple phyla in soil. *Acidobacteria* decreased from 31% of total soil bacteria before manure application to 4% at 108 DAM. *Verrucomicrobia* also decreased from 12% before manure application to 5% 108 DAM (Supplementary Table 1). *Acidobacteria* relative abundances in non-manured control soil columns increased slightly from 26 to 29% 108 days into the experiment. Additionally, *Verrucomicrobia* relative abundances increased in non-manured control columns from 8 to 11% by the end of the experiment. OTUs classified within *Proteobacteria* were enriched after manure application, rising from 12 to 35% 108 DAM, while relative abundances in non-manured control soils decreased from 18 to 12%. *Firmicutes* also increased from <1% of total bacteria in soil prior to manure application to 10% 108 DAM. *Bacteroidetes* increased after manure application from 2% before manure application to 28% 59 DAM, but subsequently decreased to 14% by 108 DAM. *Firmicutes* and *Bacteroidetes* relative abundances remained below 3% in non-manured control soils over the course of the experiment.

Similar shifts as observed in soils were also observed in drainage water over the course of the experiment. Enrichment of *Actinobacteria, Bacteroidetes, Firmicutes, Proteobacteria*, and *Verrucomicrobia* were observed between samples taken 10 DAM and 24 DAM. While *Actinobacteria*, *Proteobacteria*, and *Firmicutes* relative abundances were greatest in soil at 108 DAM, their proportions in drainage began to decrease by 59 DAM. An exception to similar trends between soil and waters was increase of *Verrucomicrobia* proportions over time in waters which decreased in soils.

To further understand the impact of manure additions on soil microbial communities, we extended our comparison of differences of observed phyla (Figure 2 and Supplementary Tables 1, 2) to the distribution of species as defined by OTUs. Our approach to assessing the impact of manure additions consisted of two methods. Firstly, we identified the OTUs which were enriched due to manure amendments (MSOs), or significantly greater in abundance (*p* < 0.05) compared to pre-manured soils (manure last applied 525 days prior) using rarified sample reads in Deseq2 (v. 3.8) (Love et al., 2014). Next, among these MSOs, we identified the MDOs, or the OTUs which originally were associated with manure (greater than 13%) and detected at only low abundance in soils [less than 2.8 × 10^-5^%]. Consequently, in the context of this study, the MDOs are a subset of MSOs which have a high probability of originating from the manure amendment.

**FIGURE 3:**
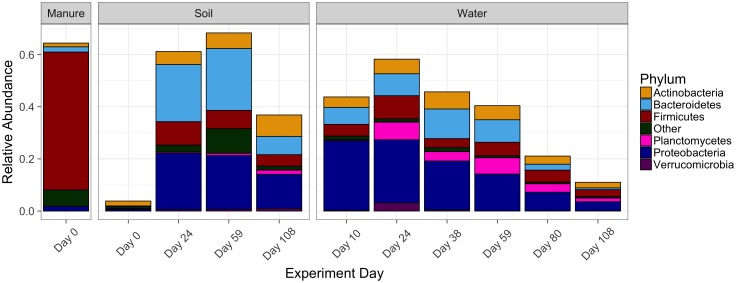
Average relative abundances of MSOs in soil and water. OTUs were classified as manure stimulated if abundances were significantly greater (*p* < 0.05) in soil 24 DAM when compared to pre-manure application soil samples.

In total, we identified the enrichment of 250 MSOs (Supplementary Table 3). In manure, the MSOs comprised 64% of the microbial community and in soils, these MSOs were present at low relative abundances and comprised 3.8% of soil microbial communities. After manure amendment, these MSOs comprised 61% of the total microbial community (average) in soil 24 DAM and 68% in soil 59 DAM (Figure 3). By 108 DAM, the MSOs decreased in manure-amended soils to 37%, though relative abundances were still elevated when compared to their presence in pre-manure application samples (0 DAM).

Overall trends in the MSO relative abundances in drainage varied slightly from those identified in corresponding soil samples. The MSOs averaged 58% of OTUs in artificial drainage 24 DAM (Figure 3). However, unlike in soils, average relative abundance of the MSOs decreased in drainage collected 59 DAM to 40%. Additionally, the set of MSOs sharply decreased in drainage to 11% on average of by 108 DAM application. Overall, in both soil and drainage, MSOs were observed to persist throughout this experiment.

**FIGURE 4:**
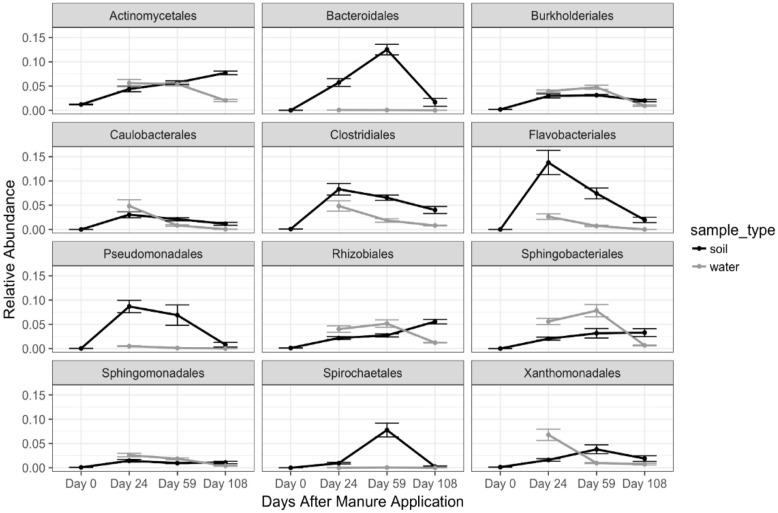
Relative abundances of bacterial OTUs in soil and water which significantly increased in soil after manure application grouped at the order level. Error bars represent standard deviations of mean soil and water relative abundances. Significant differences were determined using the function Deseq2. Day 0 soil samples were collected prior to manure application.

The majority of the identified MSOs were most closely similar to sequences belonging to 12 different orders of bacteria (Figure 4). MSOs belonging to *Proteobacteria* were most prevalent, followed by *Bacteroidetes*, *Firmicutes*, *Actinobacteria*, and *Spirochaetes*. Relative abundances of sequences matching nine of the 12 orders began to decrease by day 108. *Burkholderiales, Caulobacterales, Clostridiales, Flavobacteriales*, *Pseudomonadales*, and *Sphingomonadales* continuously decreased in manure treated soil over the course of the experiment. However, *Bacteroidales, Spirochaetales* and *Xanthomonadales* reached their maximum relative abundances in manure treated soil 59 DAM before decreasing in samples taken 108 DAM. Relative abundances of the remaining three orders continued to increase in manured soil samples 109 DAM. Although the relative abundances of certain manure stimulated orders in soil remained above proportions identified in pre-manured soil samples by 108 DAM, relative abundances of all 12 orders in drainage decreased by 108 DAM or remained consistently negligible over the course of the experiment.

The 12 orders which represented the majority of MSOs in soil also displayed varied temporal responses in drainage waters (Figure 4). Similar trends in soil and drainage water relative abundances over time were recognized in *Burkholderiales, Caulobacterales, Clostridiales, Flavobacteriales*, and *Sphingomonadales*. Trends in relative abundances of *Actinomycetales, Rhizobiales, Sphingobacteriales*, and *Xanthomonadales* in drainage water differed from those identified in soil. Relative abundances of these four orders were initially greater in drainage than soil, but decreased by 108 DAM, while in soil their proportions continued to increase. Although, *Bacteroidales, Pseudomonadales*, and *Spirochaetales* relative abundances increased in soil following manure application, very few sequences matching these orders were identified in drainage. Variable physical and chemical properties within these 12 orders of bacteria were likely responsible for differential attenuation and transport patterns over the course of our experiment.

**FIGURE 5:**
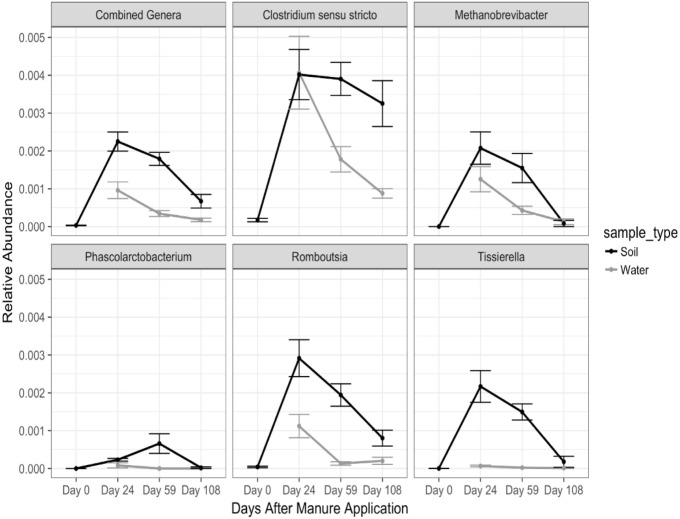
Relative abundances of manure-specific genera (MDO) in soil and drainage which significantly increased in soil after manure application. Error bars represent standard deviations of mean soil and water relative abundances. Day 0 soil samples were collected prior to manure application.

In order to identify and track bacterial populations directly introduced into soil and drainage through manure application, we next identified MDOs, which were considered a set of core bacteria specific to the swine manure and not abundant in soils. MDOs were specifically required to be identified at least 25 times in each manure sample and contained significantly greater abundance in manure compared to soil which had not received a manure application in over 30 years (*p* < 0.05). Application of these criteria resulted in five MDOs representative of: *Clostridium sensu stricto, Methanobrevibacter, Phascolarctobacterium, Romboutsia*, and *Tissierella* (Figure 5). Four of the MDOs fall within the phylum *Firmicutes*, while *Methanobrevibacter* is classified within the *Euryarchaeota* phylum. In manure samples, the five genera represent 13% of the total microbial community.

Relative abundance of the MDOs all began to decrease in soil samples 24 DAM, except *Phascolarctobacterium*, whose relative abundance peaked at 59 DAM. Additionally, all MDOs except for *Clostridium sensu stricto* returned close to pre-manure application relative abundances in soil at 108 DAM. Relative abundances of *Clostridium sensu stricto, Methanobrevibacter*, and *Romboutsia* steadily decreased in drainage samples over the course of the experiment. Conversely, *Tissierella* and *Phascolarctobacterium* were rarely identified in drainage samples. Although relative abundances of *Clostridium sensu stricto* were noticeably lower in drainage water samples when compared to soil at 108 DAM, sequences matching this genus were present in water at this time.

## Discussion

The use of soil columns in our experiment allowed us to directly assess the impacts of manure amendment on microbial communities in soil and drainage waters. Over the course of the 108-day experiment, we observed initial shifts (24 DAM) in microbial communities in soils and waters which subsequently decreased or returned to pre-manure application proportions (108 DAM). The enrichment of bacteria after manure amendments could be the direct result of manure amendment through the displacement of native soil bacteria by manure-borne bacteria or enrichment of native bacteria responding to manure available nutrients. Consequently, we defined two groups representative of manure associated communities based on their enrichment in soil and water samples (MSOs) and their likely origination from manure (MDOs).

Among MSOs, we observed short term increases in *Burkholderiales, Clostridales, Pseudomonadales*, and *Xanthomonadales* following manure incorporation, and these results are consistent with previous studies identifying these bacterial orders responding to manure amendment (Ding et al., 2014; Riber et al., 2014). Additionally, we observed that *Actinomycetales*, *Rhizobiales*, and *Sphingobacteriales* were also consistently enriched in manure-amended soils over the course of our experiment. *Actinomycetales* have also been previously identified as a dominant order associated with manure-based pyrogenic organic matter (Dai et al., 2017). Elevated levels of families within the order *Actinomycetales* have additionally been observed to remain 130 days following incorporation of dairy cow manure to soils, though in the same study, *Rhizobiales* associated families began to decrease prior to the end of the experiment (Udikovic-Kolic et al., 2014). These differences may be attributed to variations in interactions of native soil communities to the manure type and management histories of both the soils and the animals.

The MDOs identified in this study have previously been identified as abundant in swine gut or fecal microbiomes and include *Clostridium sensu stricto*, *Methanobrevibacter*, *Tissierella*, *Phascolarctobacterium*, and *Romboutsia* (Leser et al., 2002; Miller and Lin, 2002; Cotta et al., 2003; Ziemer, 2013; Tran et al., 2018). Sequences associated with *Tissierella* have previously been cited as one of few highly abundant OTUs in manure retrieved from a swine lagoon (Goh et al., 2009). *Clostridium sensu stricto* has previously been observed to also significantly increase in surface waters after fall manure application in an agriculturally dominated watershed (Rieke et al., 2018b). The Archaeal genus, *Methanobrevibacter*, known to colonize swine intestinal tracts, has also been identified in swine pens, swine waste storage pits, and anaerobic biodigesters (Peu et al., 2006; Ike et al., 2010; Da Silva et al., 2015). Overall, these results suggest that these MDOs are indicative of the presence and enrichment of swine manure microbial communities in environmental samples. While these genera have been identified in the swine gut microbiome and areas impacted by swine waste, the majority of the categorized strains within the five genera are not known to be pathogenic to humans. Additionally, it is notable that all five MDOs are obligate anaerobes. While soil is known to harbor obligate anaerobes, manure additions likely increase the number of anaerobic niche communities. However, additional test must be conducted before determining if sequences matching MDOs persist as live cells, spores, or relic DNA.

Our study identifies specific species that can be used to track swine manure impacts, and this approach has been used by others previously (Kildare et al., 2007; Okabe et al., 2007; Savichtcheva et al., 2007). In contrast to these previous studies, which often use a single species or oligotype to track manure impacts, we use five MDOs to assess the impacts of applying manure to soils. In our study, we observed the peak relative abundances of the five MDOs in soil to comprise between 0.1 and 0.5% of microbial populations. While these abundances are low fractions of the total microbial community, the MDOs were consistently detected in all manure-amended samples, further evidence for their usage in surveillance of manure-associated impacts. A key challenge to large-scale monitoring of environmental samples and manure impact is the cost and throughput of approaches to detect and quantify risks associated with manure applications. Notably, assuming 10^9^ and 10^11^ organisms per gram manure (Cotta et al., 2003), the proportions of MDOs should be detectable through qPCR methods, suggesting that development of species-specific primers could also expand the detection of these genes from phylogenetic marker sequencing to direct target approaches.

In addition to identifying manure source tracking targets, this study expanded knowledge regarding how incorporation of organic amendments in agricultural soils affect existing microbial communities. Simulation of swine manure application in the form of bands brings native soil bacteria in direct contact with available organic matter, similar to crop residue integration. Organic carbon and nitrogen additions in the form of manure or plant residue added into agricultural soils have resulted in similar increases in *Actinobacteria, Bacteroidetes*, *Proteobacteria*, and *Firmicutes* (Pascault et al., 2013; Chávez-Romero et al., 2016; Leclercq et al., 2016). However, depending on the nutrient matrix and incorporation method, certain groups of native soil microbial community members are capable of outcompeting each other. Along with organic incorporation methodology, tillage regiments have also shown to impact microbial community structure (Figuerola et al., 2012; Sipilä et al., 2012).

Soil management history response (chisel plow tillage versus no-till) had little response to manure application in our study when compared to other independent variables. However, this result may not accurately portray field managements’ influence on microbial communities due to discrepancies between our experimental design and true field conditions. Removal of the top 15 cm of soil for manure application in both no-till and chisel plow likely disrupted differences in soil structure. The manure incorporation zone disrupted any aggregation formed under no-till conditions, creating similar drainage flow paths between the chisel plow and no-till soils. Identifying the magnitude of microbial response to soil management strategies is important due to bacteria’s potential to transfer through the soil profile and into surrounding drainage waters.

Our study was the first to incorporate simulated rainfall application on soils following manure application in order to better understand bacterial community transport through the soil profile. We identified increases in the relative abundance of members within *Actinobacteria*, *Bacteroides, Firmicutes*, and *Proteobacteria* phyla in both soil and artificial drainage. Inflated relative abundances of certain groups of bacteria following manure application, common both to soil and water microbial communities, suggest the stimulated populations are easily detached from soil–manure interaction zones during matrix flow events. Once transported to a stream environment, little is currently known regarding potential survival rates of these bacterial populations and is an opportunity for further study.

Overall, the observed decreases in the majority of gene relative abundances associated with MSOs and MDOs in soil and water over the course of the experiment demonstrate agricultural soil’s natural ability to adapt to changing physical and chemical soil characteristics following organic matter additions. Swine manure is commonly injected late-fall once soil temperatures drop below 10°C or early spring after soil thaws in the Midwestern United States. Our experimental design closely mimics spring manure application timing where manure is applied shortly before typical artificial drainage seasons. Results from our study expanded our knowledge of microbial constituents that are released from the soil profile into drainage waters. This study represents an important initial characterization of this system and is a model of more complex dynamics in the natural environment. It is important for subsequent studies to expand on this effort at larger environmental scales to better characterize broadly representative bacteria indicative of potential manure presence in agroecosystems.

## Author Contributions

ER, TM, MS, FY, and AH designed, performed, and analyzed the research and wrote the paper.

## Conflict of Interest Statement

The authors declare that the research was conducted in the absence of any commercial or financial relationships that could be construed as a potential conflict of interest.
