# Supplementary material for: Temporal Dynamics of Bacterial Communities in Soil and Leachate Water After Swine Manure Application
Source: Front Microbiol. 2018 Dec 21;9:3197. doi: 10.3389/fmicb.2018.03197 (PMC6309816; doi:10.3389/fmicb.2018.03197)
Supplement: Supplementary file 1 [file Data_Sheet_1.docx]

**Temporal dynamics of bacterial communities in soil and leachate water after swine manure application Supplemental Information**

E.L. Rieke^1*^, M.L. Soupir^1^, T.B. Moorman^2^, F. Yang^1^, A. Howe^1^

^1^ Agricultural and Biosystems Engineering, Iowa State University, Ames, IA, USA.

^2^ National Laboratory for Agriculture and the Environment, USDA-ARS, Ames, IA, USA


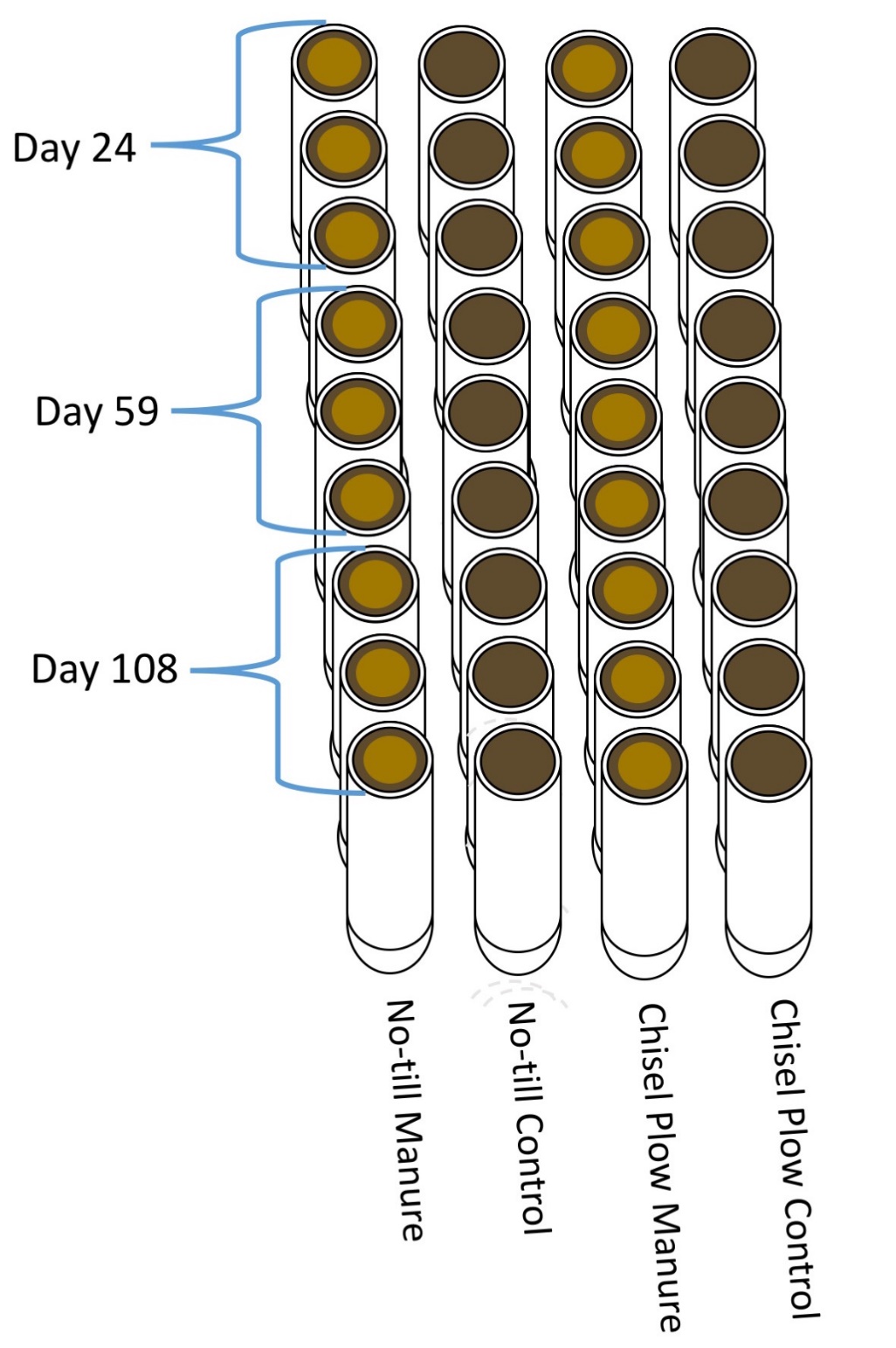


Supplementary Figure 1: Column experimental design. Columns were extracted from plots maintained under four different treatments: chisel plow non-manured control, chisel plow manure treated, no-till non-manured control and no-till manure treated. Three columns were destructed 24, 59 and 108 days following manure application.


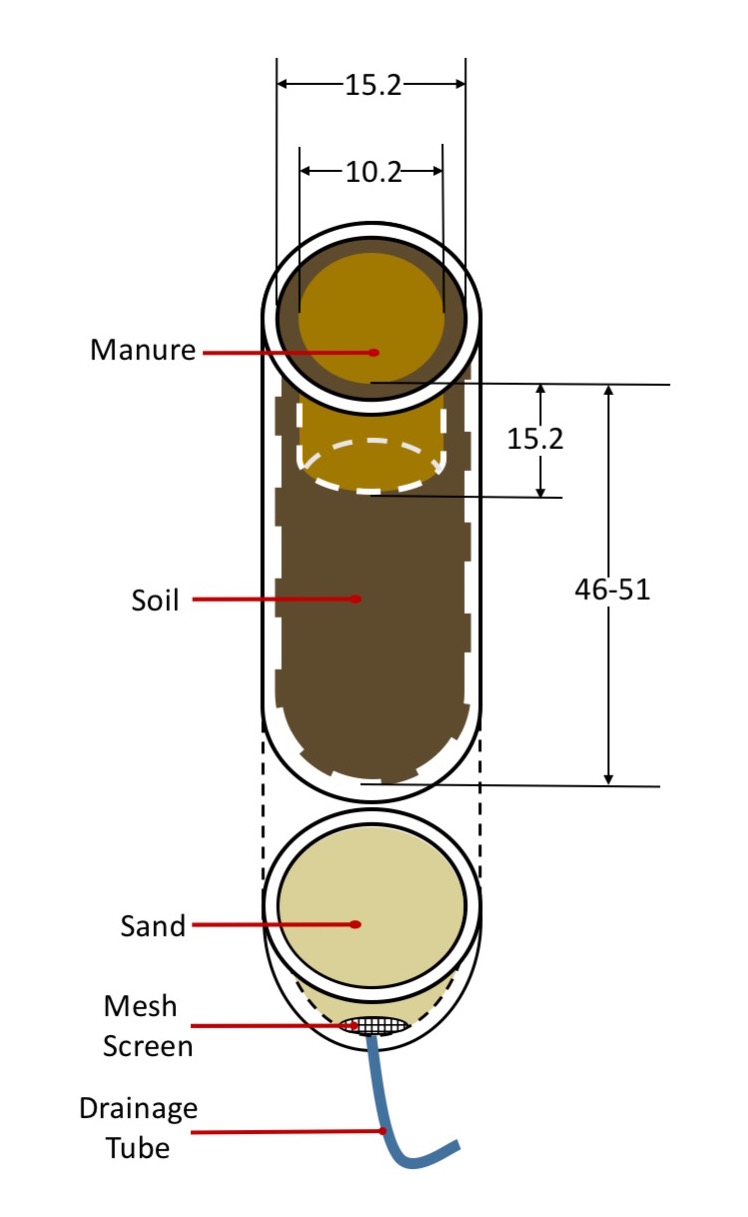


Supplementary Figure 2: Exploded manured column construction diagram. All measurements are reported in cm: 60.96 cm long, 15.24 cm diameter PVC pipes were pushed into the ground and extracted using a Giddings probe. The actual depth of the soil ranged in the pipes ranged from 46-51 cm. The 15.24 cm diameter end caps were attached to the bottom of the pipes to prevent soil loss from the bottom of the columns. A single hole was drilled in the middle of the cap to insert 0.64 diameter plastic tubing to collect drainage. The cap was then filled with ASTM 20-30 Test Sand, creating a sand-soil interface when attaching the caps. A small piece of 1.4 mm mesh was glued to the inside of the cap to prevent sand loss in in drainage.

Supplementary Table 1: Average relative abundances and standard deviations of major phyla in manure derived effluent microbial communities

|  | Average Relative Abundance ± Standard Deviation | | | | | |
| --- | --- | --- | --- | --- | --- | --- |
| Phylum | Day 10 | Day 24 | Day 38 | Day 59 | Day 80 | Day 108 |
| Acidobacteria | 0.0028 ± 0.002 | 0.0031 ± 0.0017 | 0.003 ± 0.0009 | 0.0068 ± 0.0035 | 0.005 ± 0.0025 | 0.0567 ± 0.0225a |
| Actinobacteria | 0.0424 ± 0.0271 | 0.0651 ± 0.028 | 0.0767 ± 0.0125 | 0.0642 ± 0.0174 | 0.0395 ± 0.0201 | 0.0495 ± 0.0152 |
| Bacteroidetes | 0.1354 ± 0.0409 | 0.1381 ± 0.0431 | 0.1741 ± 0.0904 | 0.1802 ± 0.0581 | 0.1109 ± 0.0745 | 0.0524 ± 0.0128a |
| candidate division WPS-1 | 0.0002 ± 0.0002 | 0.0003 ± 0.0003 | 0.0001 ± 0.0001 | 0.0002 ± 0.0002 | 0.0007 ± 0.0007 | 0.0157 ± 0.0071a |
| Chloroflexi | 0.0022 ± 0.0014 | 0.0054 ± 0.0035 | 0.0064 ± 0.0024 | 0.0113 ± 0.0052 | 0.0087 ± 0.0058 | 0.0245 ± 0.0122a |
| Cyanobacteria/Chloroplast | 0.0014 ± 0.0028 | 0.0011 ± 0.0022 | 0.0191 ± 0.024 | 0.0304 ± 0.0106 | 0.0079 ± 0.006 | 0.002 ± 0.0023 |
| Euryarchaeota | 0.0175 ± 0.0097 | 0.0129 ± 0.0064 | 0.01 ± 0.0082 | 0.0081 ± 0.0042 | 0.0051 ± 0.0034 | 0.0027 ± 0.0017a |
| Firmicutes | 0.0641 ± 0.026 | 0.1069 ± 0.023 | 0.0445 ± 0.0277 | 0.0669 ± 0.0316 | 0.0618 ± 0.0237 | 0.039 ± 0.0145 |
| Planctomycetes | 0.0075 ± 0.0063 | 0.0815 ± 0.0365 | 0.0655 ± 0.0228 | 0.1016 ± 0.0361 | 0.0753 ± 0.045 | 0.0857 ± 0.0144 |
| Proteobacteria | 0.4404 ± 0.2017 | 0.3277 ± 0.0975 | 0.3874 ± 0.0811 | 0.3822 ± 0.0548 | 0.3033 ± 0.0503 | 0.213 ± 0.0517a |
| Spirochaetes | 0.0059 ± 0.0032 | 0.0003 ± 0.0002 | 0.0062 ± 0.0124 | 0.0005 ± 0.0005 | 0 ± 0.0001 | 0 ± 0a |
| unclassified_Bacteria | 0.0126 ± 0.0073 | 0.0132 ± 0.0047 | 0.0445 ± 0.0336 | 0.0399 ± 0.0237 | 0.0776 ± 0.0487 | 0.1011 ± 0.0404 |
| Verrucomicrobia | 0.2463 ± 0.2357 | 0.2322 ± 0.1739 | 0.1359 ± 0.0522 | 0.0909 ± 0.0218 | 0.2938 ± 0.112 | 0.3288 ± 0.1733 |

a Denotes phyla whose relative abundances significantly differ between Day 10 and Day 108 using Wilcoxon Ranked Sum Test (p 0.01)

Supplementary Table 2: Average relative abundances and standard deviations of major phyla in manure treated soil microbial communities

|  | Average Relative Abundance ± Standard Deviation | | | |
| --- | --- | --- | --- | --- |
| phylum | Day 0 | Day 24 | Day 59 | Day 108 |
| Acidobacteria | 0.3057 ± 0.0564 | 0.1109 ± 0.0804 | 0.0482 ± 0.0329 | 0.0478 ± 0.0299 |
| Actinobacteria | 0.0926 ± 0.0271 | 0.0875 ± 0.0251 | 0.0814 ± 0.0157 | 0.1249 ± 0.0202 |
| Bacteroidetes | 0.0229 ± 0.0119 | 0.2296 ± 0.1091 | 0.2788 ± 0.0423 | 0.1667 ± 0.0524 |
| candidate division WPS-1 | 0.0677 ± 0.0165 | 0.0255 ± 0.0173 | 0.0101 ± 0.0078 | 0.018 ± 0.0127 |
| Chloroflexi | 0.0511 ± 0.0093 | 0.0225 ± 0.0155 | 0.0126 ± 0.0038 | 0.0211 ± 0.0081 |
| Cyanobacteria/Chloroplast | 0.0001 ± 0.0001 | 0 ± 0.0001 | 0 ± 0 | 0 ± 0.0001 |
| Euryarchaeota | 0.0005 ± 0.0005 | 0.004 ± 0.0018 | 0.0038 ± 0.0016 | 0.0018 ± 0.0019 |
| Firmicutes | 0.0073 ± 0.004 | 0.0977 ± 0.0404 | 0.0806 ± 0.0179 | 0.0494 ± 0.0206 |
| Planctomycetes | 0.074 ± 0.0142 | 0.0331 ± 0.0195 | 0.0287 ± 0.0147 | 0.0639 ± 0.0214 |
| Proteobacteria | 0.1246 ± 0.0334 | 0.2684 ± 0.0689 | 0.3061 ± 0.067 | 0.3899 ± 0.0509 |
| Spirochaetes | 0.0001 ± 0.0002 | 0.0099 ± 0.0053 | 0.0781 ± 0.0454 | 0.0023 ± 0.0044 |
| Verrucomicrobia | 0.1223 ± 0.0156 | 0.0498 ± 0.0317 | 0.034 ± 0.0161 | 0.0607 ± 0.0191 |

a Denotes phyla whose relative abundances significantly differ between Day 10 and Day 108 using Wilcoxon Ranked Sum Test (p 0.01).

Supplementary Table 3: Manure Stimulated OTUs which were enriched due to manure amendments (MSOs), or significantly greater in abundance (p<0.05) compared to pre-manured soils (manure last applied 525 days prior).

| OTU | Genus | Order |
| --- | --- | --- |
| OTU_26567 | *Acholeplasma* | *Acholeplasmatales* |
| OTU_1042 | *Pseudonocardia* | *Actinomycetales* |
| OTU_11074 | *Arthrobacter* | *Actinomycetales* |
| OTU_11319 | *Marmoricola* | *Actinomycetales* |
| OTU_1150 | *Corynebacterium* | *Actinomycetales* |
| OTU_2259 | *unclassified_Microbacteriaceae* | *Actinomycetales* |
| OTU_2329 | *Marmoricola* | *Actinomycetales* |
| OTU_2470 | *Frondihabitans* | *Actinomycetales* |
| OTU_2892 | *Mycobacterium* | *Actinomycetales* |
| OTU_3487 | *unclassified_Micromonosporaceae* | *Actinomycetales* |
| OTU_4034 | *unclassified_Microbacteriaceae* | *Actinomycetales* |
| OTU_4046 | *unclassified_Intrasporangiaceae* | *Actinomycetales* |
| OTU_4144 | *Actinomyces* | *Actinomycetales* |
| OTU_4245 | *Corynebacterium* | *Actinomycetales* |
| OTU_4323 | *unclassified_Actinomycetaceae* | *Actinomycetales* |
| OTU_4371 | *Arthrobacter* | *Actinomycetales* |
| OTU_4428 | *Aeromicrobium* | *Actinomycetales* |
| OTU_4468 | *Nocardioides* | *Actinomycetales* |
| OTU_4475 | *Agromyces* | *Actinomycetales* |
| OTU_4523 | *Yaniella* | *Actinomycetales* |
| OTU_4679 | *unclassified_Geodermatophilaceae* | *Actinomycetales* |
| OTU_4727 | *Dactylosporangium* | *Actinomycetales* |
| OTU_4783 | *Dactylosporangium* | *Actinomycetales* |
| OTU_4916 | *unclassified_Microbacteriaceae* | *Actinomycetales* |
| OTU_5022 | *Dietzia* | *Actinomycetales* |
| OTU_5122 | *Rhodococcus* | *Actinomycetales* |
| OTU_5501 | *Actinoplanes* | *Actinomycetales* |
| OTU_5608 | *unclassified_Micromonosporaceae* | *Actinomycetales* |
| OTU_6455 | *Agromyces* | *Actinomycetales* |
| OTU_6698 | *Rhodococcus* | *Actinomycetales* |
| OTU_7508 | *Nocardia* | *Actinomycetales* |
| OTU_4537 | *unclassified_Anaerolineaceae* | *Anaerolineales* |
| OTU_12051 | *Lysinibacillus* | *Bacillales* |
| OTU_12130 | *Bacillus* | *Bacillales* |
| OTU_12328 | *unclassified_Bacillales* | *Bacillales* |
| OTU_2296 | *Lysinibacillus* | *Bacillales* |
| OTU_4013 | *Bacillus* | *Bacillales* |
| OTU_4806 | *Sporosarcina* | *Bacillales* |
| OTU_6280 | *unclassified_Planococcaceae* | *Bacillales* |
| OTU_1174 | *unclassified_Marinilabiliaceae* | *Bacteroidales* |
| OTU_14120 | *unclassified_Bacteroidales* | *Bacteroidales* |
| OTU_1990 | *unclassified_Prevotellaceae* | *Bacteroidales* |
| OTU_3873 | *unclassified_Porphyromonadaceae* | *Bacteroidales* |
| OTU_4298 | *Geofilum* | *Bacteroidales* |
| OTU_4598 | *unclassified_Bacteroidales* | *Bacteroidales* |
| OTU_4920 | *Petrimonas* | *Bacteroidales* |
| OTU_4925 | *Petrimonas* | *Bacteroidales* |
| OTU_4975 | *Petrimonas* | *Bacteroidales* |
| OTU_5008 | *Proteiniphilum* | *Bacteroidales* |
| OTU_5184 | *unclassified_Porphyromonadaceae* | *Bacteroidales* |
| OTU_5289 | *Petrimonas* | *Bacteroidales* |
| OTU_12046 | *unclassified_Alcaligenaceae* | *Burkholderiales* |
| OTU_12093 | *Pusillimonas* | *Burkholderiales* |
| OTU_12112 | *unclassified_Alcaligenaceae* | *Burkholderiales* |
| OTU_14600 | *Alcaligenes* | *Burkholderiales* |
| OTU_1577 | *Curvibacter* | *Burkholderiales* |
| OTU_1783 | *Massilia* | *Burkholderiales* |
| OTU_1951 | *Candidimonas* | *Burkholderiales* |
| OTU_1987 | *Herbaspirillum* | *Burkholderiales* |
| OTU_1996 | *Oligella* | *Burkholderiales* |
| OTU_2439 | *Castellaniella* | *Burkholderiales* |
| OTU_3956 | *Paenalcaligenes* | *Burkholderiales* |
| OTU_3958 | *unclassified_Alcaligenaceae* | *Burkholderiales* |
| OTU_3966 | *Pusillimonas* | *Burkholderiales* |
| OTU_5516 | *Comamonas* | *Burkholderiales* |
| OTU_7190 | *Achromobacter* | *Burkholderiales* |
| OTU_10185 | *Brevundimonas* | *Caulobacterales* |
| OTU_12042 | *Brevundimonas* | *Caulobacterales* |
| OTU_3838 | *Brevundimonas* | *Caulobacterales* |
| OTU_6739 | *Caulobacter* | *Caulobacterales* |
| OTU_7522 | *Caulobacter* | *Caulobacterales* |
| OTU_8178 | *Brevundimonas* | *Caulobacterales* |
| OTU_4162 | *Halothiobacillus* | *Chromatiales* |
| OTU_1156 | *Syntrophomonas* | *Clostridiales* |
| OTU_1183 | *Clostridium sensu stricto* | *Clostridiales* |
| OTU_26483 | *Terrisporobacter* | *Clostridiales* |
| OTU_26484 | *Tissierella* | *Clostridiales* |
| OTU_26487 | *Romboutsia* | *Clostridiales* |
| OTU_26489 | *Alkaliphilus* | *Clostridiales* |
| OTU_26490 | *Tissierella* | *Clostridiales* |
| OTU_26494 | *Tissierella* | *Clostridiales* |
| OTU_26498 | *Guggenheimella* | *Clostridiales* |
| OTU_26500 | *Tissierella* | *Clostridiales* |
| OTU_26507 | *unclassified_Clostridiales* | *Clostridiales* |
| OTU_26508 | *Tissierella* | *Clostridiales* |
| OTU_26513 | *Tissierella* | *Clostridiales* |
| OTU_26708 | *Alkaliphilus* | *Clostridiales* |
| OTU_26714 | *Sedimentibacter* | *Clostridiales* |
| OTU_3835 | *Clostridium sensu stricto* | *Clostridiales* |
| OTU_3867 | *Clostridium sensu stricto* | *Clostridiales* |
| OTU_4135 | *Clostridium sensu stricto* | *Clostridiales* |
| OTU_4140 | *unclassified_Clostridiales* | *Clostridiales* |
| OTU_4141 | *Clostridium XlVa* | *Clostridiales* |
| OTU_4143 | *unclassified_Clostridiales* | *Clostridiales* |
| OTU_4146 | *unclassified_Ruminococcaceae* | *Clostridiales* |
| OTU_4147 | *unclassified_Ruminococcaceae* | *Clostridiales* |
| OTU_4148 | *unclassified_Ruminococcaceae* | *Clostridiales* |
| OTU_4149 | *unclassified_Lachnospiraceae* | *Clostridiales* |
| OTU_4155 | *unclassified_Eubacteriaceae* | *Clostridiales* |
| OTU_4174 | *Clostridium sensu stricto* | *Clostridiales* |
| OTU_4175 | *unclassified_Clostridiaceae 1* | *Clostridiales* |
| OTU_4177 | *Clostridium III* | *Clostridiales* |
| OTU_4279 | *unclassified_Clostridiales* | *Clostridiales* |
| OTU_4872 | *unclassified_Clostridiales* | *Clostridiales* |
| OTU_5213 | *Tissierella* | *Clostridiales* |
| OTU_26493 | *Senegalimassilia* | *Coriobacteriales* |
| OTU_14545 | *unclassified_Cyclobacteriaceae* | *Cytophagales* |
| OTU_5412 | *Mariniradius* | *Cytophagales* |
| OTU_5573 | *Desulfocella* | *Desulfobacterales* |
| OTU_24752 | *Desulfuromonas* | *Desulfuromonadales* |
| OTU_2442 | *Enterobacter* | *Enterobacteriales* |
| OTU_2468 | *Yersinia* | *Enterobacteriales* |
| OTU_2664 | *Turicibacter* | *Erysipelotrichales* |
| OTU_3967 | *Fibrobacter* | *Fibrobacterales* |
| OTU_12047 | *Aequorivita* | *Flavobacteriales* |
| OTU_12048 | *Chryseobacterium* | *Flavobacteriales* |
| OTU_12120 | *unclassified_Cryomorphaceae* | *Flavobacteriales* |
| OTU_12314 | *Brumimicrobium* | *Flavobacteriales* |
| OTU_12341 | *Aequorivita* | *Flavobacteriales* |
| OTU_12362 | *unclassified_Flavobacteriaceae* | *Flavobacteriales* |
| OTU_12723 | *unclassified_Flavobacteriaceae* | *Flavobacteriales* |
| OTU_14504 | *Arenibacter* | *Flavobacteriales* |
| OTU_14541 | *Flavobacterium* | *Flavobacteriales* |
| OTU_14546 | *unclassified_Flavobacteriaceae* | *Flavobacteriales* |
| OTU_1983 | *Flavobacterium* | *Flavobacteriales* |
| OTU_3877 | *unclassified_Flavobacteriaceae* | *Flavobacteriales* |
| OTU_4946 | *unclassified_Flavobacteriaceae* | *Flavobacteriales* |
| OTU_5356 | *Gelidibacter* | *Flavobacteriales* |
| OTU_5859 | *Flavobacterium* | *Flavobacteriales* |
| OTU_6754 | *Salinirepens* | *Flavobacteriales* |
| OTU_6895 | *Myroides* | *Flavobacteriales* |
| OTU_3458 | *Gaiella* | *Gaiellales* |
| OTU_3654 | *Gaiella* | *Gaiellales* |
| OTU_1221 | *Gemmatimonas* | *Gemmatimonadales* |
| OTU_12659 | *Gemmatimonas* | *Gemmatimonadales* |
| OTU_6216 | *Gemmatimonas* | *Gemmatimonadales* |
| OTU_4131 | *Atopostipes* | *Lactobacillales* |
| OTU_4294 | *Lactobacillus* | *Lactobacillales* |
| OTU_1132 | *Methanobrevibacter* | *Methanobacteriales* |
| OTU_1158 | *Methanobrevibacter* | *Methanobacteriales* |
| OTU_886 | *Methanosphaera* | *Methanobacteriales* |
| OTU_15207 | *Anaeromyxobacter* | *Myxococcales* |
| OTU_5285 | *Polyangium* | *Myxococcales* |
| OTU_5712 | *Chondromyces* | *Myxococcales* |
| OTU_3514 | *Nitrososphaera* | *Nitrososphaerales* |
| OTU_12395 | *Marinospirillum* | *Oceanospirillales* |
| OTU_6429 | *Oligoflexus* | *Oligoflexales* |
| OTU_1008 | *Singulisphaera* | *Planctomycetales* |
| OTU_1381 | *Singulisphaera* | *Planctomycetales* |
| OTU_4278 | *unclassified_Planctomycetaceae* | *Planctomycetales* |
| OTU_865 | *Singulisphaera* | *Planctomycetales* |
| OTU_867 | *Aquisphaera* | *Planctomycetales* |
| OTU_868 | *Singulisphaera* | *Planctomycetales* |
| OTU_892 | *Singulisphaera* | *Planctomycetales* |
| OTU_9167 | *unclassified_Planctomycetaceae* | *Planctomycetales* |
| OTU_924 | *Aquisphaera* | *Planctomycetales* |
| OTU_12648 | *Pseudomonas* | *Pseudomonadales* |
| OTU_16379 | *Pseudomonas* | *Pseudomonadales* |
| OTU_1981 | *Acinetobacter* | *Pseudomonadales* |
| OTU_2591 | *Pseudomonas* | *Pseudomonadales* |
| OTU_407 | *unclassified_Pseudomonadaceae* | *Pseudomonadales* |
| OTU_4176 | *unclassified_Pseudomonadaceae* | *Pseudomonadales* |
| OTU_4411 | *Thiopseudomonas* | *Pseudomonadales* |
| OTU_4942 | *unclassified_Pseudomonadaceae* | *Pseudomonadales* |
| OTU_5344 | *Pseudomonas* | *Pseudomonadales* |
| OTU_11947 | *Devosia* | *Rhizobiales* |
| OTU_12034 | *Falsochrobactrum* | *Rhizobiales* |
| OTU_12058 | *Kaistia* | *Rhizobiales* |
| OTU_12062 | *Aquamicrobium* | *Rhizobiales* |
| OTU_12081 | *Devosia* | *Rhizobiales* |
| OTU_12109 | *Camelimonas* | *Rhizobiales* |
| OTU_2351 | *Rhodopseudomonas* | *Rhizobiales* |
| OTU_2653 | *Ochrobactrum* | *Rhizobiales* |
| OTU_4203 | *unclassified_Rhizobiaceae* | *Rhizobiales* |
| OTU_4292 | *Aminobacter* | *Rhizobiales* |
| OTU_4295 | *Microvirga* | *Rhizobiales* |
| OTU_4721 | *unclassified_Rhizobiales* | *Rhizobiales* |
| OTU_4956 | *Devosia* | *Rhizobiales* |
| OTU_5168 | *Devosia* | *Rhizobiales* |
| OTU_5241 | *Mesorhizobium* | *Rhizobiales* |
| OTU_5302 | *Devosia* | *Rhizobiales* |
| OTU_7089 | *Mesorhizobium* | *Rhizobiales* |
| OTU_7576 | *Bosea* | *Rhizobiales* |
| OTU_14614 | *Paracoccus* | *Rhodobacterales* |
| OTU_6055 | *Paracoccus* | *Rhodobacterales* |
| OTU_4361 | *unclassified_Rhodospirillales* | *Rhodospirillales* |
| OTU_26418 | *Phascolarctobacterium* | *Selenomonadales* |
| OTU_3630 | *Conexibacter* | *Solirubrobacterales* |
| OTU_3818 | *Solirubrobacter* | *Solirubrobacterales* |
| OTU_4643 | *Conexibacter* | *Solirubrobacterales* |
| OTU_5030 | *Conexibacter* | *Solirubrobacterales* |
| OTU_5590 | *Solirubrobacter* | *Solirubrobacterales* |
| OTU_6048 | *Conexibacter* | *Solirubrobacterales* |
| OTU_3412 | *Nitrolancea* | *Sphaerobacterales* |
| OTU_12037 | *Sphingobacterium* | *Sphingobacteriales* |
| OTU_12072 | *Sphingobacterium* | *Sphingobacteriales* |
| OTU_12087 | *Taibaiella* | *Sphingobacteriales* |
| OTU_12089 | *unclassified_Sphingobacteriaceae* | *Sphingobacteriales* |
| OTU_12307 | *Sphingobacterium* | *Sphingobacteriales* |
| OTU_12455 | *Pedobacter* | *Sphingobacteriales* |
| OTU_14498 | *unclassified_Sphingobacteriaceae* | *Sphingobacteriales* |
| OTU_14503 | *unclassified_Sphingobacteriaceae* | *Sphingobacteriales* |
| OTU_14507 | *unclassified_Sphingobacteriaceae* | *Sphingobacteriales* |
| OTU_2314 | *Mucilaginibacter* | *Sphingobacteriales* |
| OTU_2445 | *Pedobacter* | *Sphingobacteriales* |
| OTU_2471 | *Taibaiella* | *Sphingobacteriales* |
| OTU_3938 | *Pedobacter* | *Sphingobacteriales* |
| OTU_4628 | *unclassified_Chitinophagaceae* | *Sphingobacteriales* |
| OTU_8243 | *Pedobacter* | *Sphingobacteriales* |
| OTU_9646 | *Taibaiella* | *Sphingobacteriales* |
| OTU_12065 | *Sphingorhabdus* | *Sphingomonadales* |
| OTU_2317 | *Sphingomonas* | *Sphingomonadales* |
| OTU_2319 | *Novosphingobium* | *Sphingomonadales* |
| OTU_2457 | *Sphingopyxis* | *Sphingomonadales* |
| OTU_5927 | *Sphingobium* | *Sphingomonadales* |
| OTU_7504 | *Sphingopyxis* | *Sphingomonadales* |
| OTU_3947 | *Treponema* | *Spirochaetales* |
| OTU_3959 | *Treponema* | *Spirochaetales* |
| OTU_3960 | *Treponema* | *Spirochaetales* |
| OTU_4357 | *Treponema* | *Spirochaetales* |
| OTU_4401 | *Treponema* | *Spirochaetales* |
| OTU_4291 | *unclassified_Synergistaceae* | *Synergistales* |
| OTU_5091 | *Thermoleophilum* | *Thermoleophilales* |
| OTU_3031 | *Gp6* | *unclassified_Acidobacteria_Gp6* |
| OTU_4882 | *unclassified_Actinobacteria* | *unclassified_Actinobacteria* |
| OTU_5465 | *unclassified_Alphaproteobacteria* | *unclassified_Alphaproteobacteria* |
| OTU_27200 | *unclassified_Bacteria* | *unclassified_Bacteria* |
| OTU_3993 | *unclassified_Bacteria* | *unclassified_Bacteria* |
| OTU_545 | *unclassified_Bacteria* | *unclassified_Bacteria* |
| OTU_14525 | *unclassified_Bacteroidetes* | *unclassified_Bacteroidetes* |
| OTU_14544 | *unclassified_Bacteroidetes* | *unclassified_Bacteroidetes* |
| OTU_6956 | *WPS-1_genera_incertae_sedis* | *unclassified_candidate division WPS-1* |
| OTU_12326 | *unclassified_Deltaproteobacteria* | *unclassified_Deltaproteobacteria* |
| OTU_4442 | *unclassified_Firmicutes* | *unclassified_Firmicutes* |
| OTU_4706 | *unclassified_Gammaproteobacteria* | *unclassified_Gammaproteobacteria* |
| OTU_3874 | *unclassified_Proteobacteria* | *unclassified_Proteobacteria* |
| OTU_3823 | *unclassified_Spartobacteria* | *unclassified_Spartobacteria* |
| OTU_4213 | *Spartobacteria_genera_incertae_sedis* | *unclassified_Spartobacteria* |
| OTU_5312 | *unclassified_Spartobacteria* | *unclassified_Spartobacteria* |
| OTU_3469 | *Subdivision3_genera_incertae_sedis* | *unclassified_Subdivision3* |
| OTU_24868 | *Subdivision5_genera_incertae_sedis* | *unclassified_Subdivision5* |
| OTU_3034 | *unclassified_Thermomicrobia* | *unclassified_Thermomicrobia* |
| OTU_5034 | *unclassified_Verrucomicrobia* | *unclassified_Verrucomicrobia* |
| OTU_12033 | *Thermomonas* | *Xanthomonadales* |
| OTU_12060 | *Stenotrophomonas* | *Xanthomonadales* |
| OTU_12061 | *Stenotrophomonas* | *Xanthomonadales* |
| OTU_12129 | *Lysobacter* | *Xanthomonadales* |
| OTU_2032 | *Stenotrophomonas* | *Xanthomonadales* |
| OTU_4596 | *Luteimonas* | *Xanthomonadales* |
| OTU_6052 | *unclassified_Xanthomonadaceae* | *Xanthomonadales* |

Supplementary Table 4: Classifiers of Unique ID given to samples deposited in NCBI SRA with the following accession number: PRJNA506065

| Descriptor | Identifier | Description |
| --- | --- | --- |
| ID | A### | Unique ID |
| Manure Management | Ma | Manure applied soils |
|  | Co | Non manured control soils |
| Field Management | Cp | Chisel plow management |
|  | Nt | No till |
| Soil Sampling Time Point | S0 | Day 0 |
|  | S1 | Day 24 |
|  | S2 | Dat 59 |
|  | S3 | Day 108 |
| Simulated Rainfal Sampling Point | R1 | Day 10 |
|  | R2 | Day 24 |
|  | R3 | Day 38 |
|  | R4 | Day 59 |
|  | R5 | Day 80 |
|  | R6 | Day108 |
